# COVID-19: Pandemic of Cognitive Biases Impacting Human Behaviors and Decision-Making of Public Health Policies

**DOI:** 10.3389/fpubh.2020.613290

**Published:** 2020-11-24

**Authors:** François Lechanoine, Katlyn Gangi

**Affiliations:** ^1^Department of Neurosurgery, University Hospital Centre Grenoble Alpes, Grenoble, France; ^2^Department of Communication Studies, University of Montana, Missoula, MT, United States

**Keywords:** COVID-19 pandemic, SARS-CoV-2, cognitive biases, heuristics, human behavior, decision-making, public health policy

## Introduction

Although disasters such as wars and epidemics have plagued the planet for centuries, the ability of humanity to forget lessons learned through history is remarkable. One reason this happens is that cognitive biases, first described in 1974, challenge our rational thinking (1) and can lead us astray in our decision making. These biases are systematic and predictable errors of judgment affecting the human thought in situations of uncertainty—such as the COVID-19 pandemic.

## Main Cognitive Biases

Facing a crisis, our brain references situations we have already experienced, leading to a **belief bias**, varying individually and collectively around the world. Many Asian countries, such as South Korea or Taiwan, severely hit by the deadly SARS-CoV in 2003, appeared to have been better prepared against the novel coronavirus pandemic, applying strong measures incredibly early on in the spread of the virus. Asian authorities and population thus appealed to the belief bias coupled with the **availability bias**. In other words, the experience of the relatively recent SARS-CoV virus was available in their memories, and thus they could retrieve this information to better respond to the novel coronavirus. This availability bias resulted in the world not seriously considering the horror of the H1N1 pandemic of 1918–1920, which decimated more than 50 million of the population of the globe, simply because it is not accessible as part of recent memory.

In contrast, many western countries' authorities who had been confronted with the pandemic of Influenza A (H1N1) in 2009 have been accused of overreaction, while the death rate has now exceeded that of the seasonal flu. As such, collective memory does not seem to exceed a generation. Furthermore, the media contributing a continuous stream of often uncertain information may exacerbate these mental shortcuts being applied to this pandemic.

Moreover, the **representativeness heuristic** pushes people to overestimate the probability of low-risk events, such as becoming a victim of a terrorist attack, and underestimate high probability risks, like becoming infected with a virus during a pandemic. Additionally, diverse social environments influence differently human fears about diseases, leading to public misperceptions of risks and readily affect behaviors and subsequent decision-making (2).

The **bandwagon effect** is the tendency to do something primarily because others are doing it, rather than following one's own beliefs. The bandwagon effect and **in-group favoritism** focus our attention on people who differ from us. We perceive that disasters happening to people from other cultures, other countries, other communities (considered the out-group), cannot happen to us. One after the other, authorities underestimated the disaster occurring at their doors, leading each country to surpass the viral spread and death curves of the previous one.

Experts exhibit **overconfidence** and **exponential-growth biases**. Facing the very first cases and deaths of the current pandemic, most of the world's leading epidemiologists were wrong by a factor of at least 10 in the prediction of its evolution.

Inevitably, **hindsight bias** will erode collective memory. Whatever the number of deaths and socio-economic issues will be, no one will remember how uncertain the situation was at the beginning. Most of the authorities are already accused of mismanaging the crisis, being late and unprepared, or on the contrary by having “unnecessarily” weighed down the country's economy.

## Discussion

Cognitive biases strike every human being—even physicians. As scientists, we must strongly consider the ways in which our own judgment is affected by these cognitive biases, especially under conditions of uncertainty. They must be methodically identified and eliminated if possible. They can lead populations, experts, authorities to arrive at incorrect assessments of situations, and then engage in misguided responses. Viruses continuously test our individual and collective immunity systems but also our collective memory and behaviors. As physicians, researchers, scientists, public health authorities and organizations, we are challenged by a new crisis in our History, the SARS-Cov-2 Pandemic.

No common public health policy has been applied to fight this initially global health crisis, even within the same country. It is highly probable that the errors of judgment due to the cognitive biases of our scientific community have led or at least contributed to disorganized behaviors and decisional errors of politics with serious consequences. Scientific recommendations in such a crisis must be as unanimous as possible to facilitate public health policies and decision. Even the greatest experts should be subjected to the detection of cognitive biases by their peers, or by machines, that can learn from our mistakes. It is fair to raise the question: will have Artificial Intelligence saved lives, avoiding human cognitive biases? Should we entrust our decision-making processes to computers to protect us from ourselves? However, once these biases may have been eliminated, a strong common public health policy is still needed. Maybe the authorities and the World Health Organization could have stopped the spread of the virus early on if they had a more accurate and less biased view of the situation. Is it time to overhaul the global systems of vigilance and response to epidemics to a more efficient centralized operational system, even if it means weakening the sovereignty of each country?

## Author Contributions

FL contributed to the design, the draft of the manuscript, to data recovery, and final revision of the manuscript. KG contributed to the draft of the manuscript and English editing. All authors contributed to the article and approved the submitted version.

## Conflict of Interest

The authors declare that the research was conducted in the absence of any commercial or financial relationships that could be construed as a potential conflict of interest.
